# Community Health Workers and Health Care Delivery: Evaluation of a Women's Reproductive Health Care Project in a Developing Country

**DOI:** 10.1371/journal.pone.0075476

**Published:** 2013-09-25

**Authors:** Abdul Wajid, Franklin White, Mehtab S. Karim

**Affiliations:** 1 College of Human Medicine, Michigan State University, East Lansing, Michigan, United States of America; 2 School of Public Health and Social Policy, University of Victoria, BC, Canada; 3 Community Health and Epidemiology, Dalhousie University, NS, Canada; 4 Pacific Health & Development Sciences Inc., Victoria, BC, Canada; 5 School of Public Policy, George Mason University, Arlington, Virginia, United States of America; The University of Adelaide, Australia

## Abstract

**Background:**

As part of the mid-term evaluation of a Women's Health Care Project, a study was conducted to compare the utilization of maternal and neonatal health (MNH) services in two areas with different levels of service in Punjab, Pakistan.

**Methods:**

A cross-sectional survey was conducted to interview Married Women of Reproductive Age (MWRA). Information was collected on MWRA knowledge regarding danger signs during pregnancy, delivery, postnatal periods, and MNH care seeking behavior. After comparing MNH service utilization, the two areas were compared using a logistic regression model, to identify the association of different factors with the intervention after controlling for socio-demographic, economic factors and distance of the MWRA residence to a health care facility.

**Results:**

The demographic characteristics of women in the two areas were similar, although socioeconomic status as indicated by level of education and better household amenities, was higher in the intervention area. Consequently, on univariate analysis, utilization of MNH services: antenatal care, TT vaccination, institutional delivery and use of modern contraceptives were higher in the intervention than control area. Nonetheless, multivariable analysis controlling for confounders such as socioeconomic status revealed that utilization of antenatal care services at health centers and TT vaccination during pregnancy are significantly associated with the intervention.

**Conclusions:**

Our findings suggest positive changes in health care seeking behavior of women and families with respect to MNH. Some aspects of care still require attention, such as knowledge about danger signs and neonatal care, especially umbilical cord care. Despite overall success achieved so far in response to the Millennium Development Goals, over the past two decades decreases in maternal mortality are far from the 2015 target. This report identifies some of the key factors to improving MNH and serves as an interim measure of a national and global challenge that remains a work in progress.

## Introduction

Globally, maternal deaths decreased by 47% from 1990 to 2010. By 2010, two regions, Sub- Saharan Africa (56%) and Southern Asia (29%), together attributed about 85% of the global burden (245 000 maternal deaths) [1]. This is commonly monitored by the Maternal Mortality Ratio (MMR), the number of maternal deaths in a given time period per 100 000 live births during the same time period. The overall MMR for the world fell from 400 to 210 per 100,000, while for developing regions it fell from 440 to 240 during the same 20 year period. In 2010, the estimate for Pakistan was 260 (range of uncertainty 150–500); within the country the ratio is even higher in remote, rural and underdeveloped areas, largely due to non-availability and low utilization of maternal health services. Apart from socioeconomic factors, evidence suggests that utilization of these services also depends on timely awareness of health care needs, service availability and accessibility, quality of care, and attitude of facility staff towards clients [2]–[7].

Pakistan is a signatory to the United Nations Millennium Development Goals (MDG); the MDG-5 is to reduce MMR by three quarters and provide universal access to reproductive health by the year 2015 [8]. In an effort to improve maternal and neonatal health services, the Government of Pakistan launched at national level a community based primary health care (PHC) program, known as the National Program for Family Planning and Primary Health Care (NPFPPHC, often referred as the LHW program) in 1994 in a phased manner; the program commenced in district Rahim Yar Khan in south Punjab in 1997. Its purpose is to provide maternal, neonatal and child health services in rural and semi-urban areas. The Lady Health Worker (LHW) is the backbone of the program, who numbering approximately 100,000 nationally. The minimum qualifications for entry into LHW training are education at grade 8 level with residential status in the community from which she is recruited. She then passes through 15 months training and receives a monthly stipend. Her responsibilities include: registration of families and focusing on health related issues of Married Women of Reproductive Age (MWRA) and children; she creates awareness and provides health education on maternal and child health issues. She also provides some methods of contraception to her registered couples [9]. Apart from the LHW program, research and training projects have also focused on improving maternal health and MDG-5 [10], [11].

### Public health infrastructure in Pakistan

A remarkable shift in Pakistan’s public health infrastructure came in the 1970s when a nationwide establishment of health centers took place, providing health services in village and urban slums. These health facilities are usually based on the population of their catchment area. Primary care facilities include [12]: Health House of a Lady Health Worker, Maternal and Child Health Centers (MCHCs), Basic Health Units (BHUs) and Rural Health Centers (RHCs). Since 1994, when the LHW Program was started, health house has played a role in providing very basic health services, including Maternal, Neonatal and Child Health (MNCH) related messages, some medicines such as oral rehydration salt, anthelminthics for deworming of children, and contraceptives. These services are provided by an LHW, covering a population of approximately 1000 individuals.

Lady Health Visitors (LHVs) oversee MCHCs and provide ante-natal, normal delivery, post- natal and Family Planning (FP) services with very basic curative care for mothers and newborns. Basic Health Units are headed by a doctor assisted by a medical technician, dispenser, LHV and a *dai* (traditional birth attendant who receives tips and training usually from older females in the family; in rural communities she is mostly the preferred attendant at delivery). The scope of services of BHUs is greater than MCHC as basic curative services are also provided in addition to preventive, FP and mother and child health services. These two health facilities, MCHC and BHU, provide services during daytime. MCHCs are usually located in urban areas or some large rural areas which are far away from a BHU or RHC. The population served by a BHU ranges from 10,000–25,000. The RHCs provide round-the-clock comprehensive services, including an Operating Theater (OT) and dental services, supported by basic laboratory and radiology facilities. These centers provide comprehensive primary health services, with MNCH services usually provided by a woman medical officer.

Higher or referral level health facilities include Tehsil Headquarters Hospital (THQH) and District Headquarters Hospital (DHQH). The former is located at sub-district level providing preventive and curative services to a population of about half a million to one million. The latter serves an even larger catchment area with population ranging from one to three million. Both these facilities offer advanced level health services as specialist doctors are also appointed here, including a pediatrician and a gynecologist. These facilities also have inpatient services ranging from 40-250 beds.

### Women’s Health Project

In 1994, the same year when the national LHW program was started, an international NGO, Médecins du Monde (MDM),, began to implement a Women’s Reproductive Health Care Project in one of the Tehsils (sub-districts), of the district Rahim Yar Khan to reduce maternal deaths and thus contribute in achieving MDG-5 [13].

The project carried out various actions and activities both at community and facility level to address the three delay model: (1) delay in the decision to seek care; (2) delay in arrival at a health facility; and (3) delay in the provision of adequate care [14]. Related measures included:

- *Appointment of a woman medical officer and round-the-clock coverage of midwife/LHV at RHC Nawankot:* Usually, the government’s allocated woman medical officer position remains vacant due to security issues or inaccessibility to the health facility. Nonetheless, the presence of an experienced doctor can play a significant role in maternal and child health especially for emergency situations. To address this issue of non-availability of a woman medical officer, MDM appointed a residential woman medical officer who attended the patients during day-time and was on-call after hours. An LHV or midwife was supposed to be present in the facility round the clock.
- *Provision of diagnostic facilities at RHC Nawankot:* Apart from the usual basic laboratory and radiology services, MDM had arranged ultrasonographic services which were usually provided by a senior doctor/Head of Mission residing locally and attending the facility.
- *Training of health care staff of BHUs, RHC and THQH:* Staff members of the project area health facilities were trained to improve their technical and communication skills.
- *Promotion and Training of LHWs working for the NPFPPHC:* Most of the geographic areas of the project site did not have LHWs as the program could not find girls with a minimum of grade 8 education. To address this issue, MDM, with the approval of NPFPPHC, identified potential candidates from the uncovered areas, organized their basic education classes to bring them to a level to complete the tasks of an LHW. After completing an approved syllabus, the candidates were to satisfy the LHW program for a minimum level of education and then join as a trainee LHW.
- *Recruitment & training of Traditional Birth Attendants (TBAs):* To address the issue of shortage of care providers during pregnancy and delivery, appropriate candidates were identified at local rural areas with the highest need and trained in appropriate practices of care including maintenance of aseptic measures and early referrals in case of any emergency or delayed labor. These females are called TBAs (the terms *dais* and TBAs are used interchangeably).
- *Establishment of Village Health Committees (VHCs):* The project also established VHCs with the help of local communities who were involved in mobilization, enhancing awareness about Maternal and Neonatal Health (MNH) issues and managing transportation problems at the time of referral of obstetrical emergencies.

The three delays were addressed more specifically through the following steps:

- Promotion of awareness at family and community levels through VHCs, TBAs and LHWs;
- Transportation to health facility for emergency obstetric care by VHCs;
- Improvement in quality of care at health facilities by providing round-the-clock skilled care providers and advanced diagnostic services.

In 2001, UNFPA requested the co-authors who then were associated with the Aga Khan University, for a midterm evaluation (MTE) to guide planning of this ongoing project. This article draws from that MTE, and may be viewed as a case study of an ongoing process to improve maternal mortality not only in Pakistan, but also globally as noted in the Introduction. It is important to learn from this experience, especially as the world works towards the achievement of MDG-5.

### Objectives

1. To estimate the prevalence of utilization of different components of MNH Care services among Married Women of Reproductive Age (MWRA) in selected rural areas of Tehsil Khan Pur.
2. To determine the association of MNH Care factors with the NGO health service interventions when compared with a non-intervention area (control).

## Methodology

### Study design & Setting

This study applied a cross-sectional survey in rural areas of Tehsil Khan Pur, in September through November 2001 (the terms Tehsil and sub-district are used interchangeably). The sampling unit was the household and the study element was the MWRA living in those households. We included all MWRA living in two areas (intervention and non- intervention) with at least one delivery within the past 18 months. Individuals were excluded if they migrated to the study area (from non-study area) after January 2001 (stay period in the intervention areas less than the duration of one gestational period).

We calculated sample size for two purposes: estimation of prevalence and for association of factors with the dependent variable. Then the team chose the larger of the two estimates as the number for data collection, adjusted for design effect and non-response. After all adjustments, we took 472 as the final sample size for each area.

Afterwards, the research team calculated sample size for both areas taking prevalence of antenatal visit separately in these areas (intervention and non-intervention); using the formula for sample size calculation to estimate the prevalence of a factor [15], applying the following assumptions:

p  =  Prevalence of antenatal care in the rural areas of Pakistan at the time of sample size calculation,

B  =  Bound on error

Type I error for all areas was taken as 5% while B as 6%.

In Pakistan’s rural areas, 25–27 percent of women received antenatal care around the time when we determined sample size. Therefore, we took prevalence for the non-intervention area as 25 percent [16]. As we selected two different areas for this survey (intervention and non- intervention) and did not have data available for other specific situations, we assumed that intervention would have more than doubled the prevalence as compared with non-intervention areas; on this basis therefore we decided to take the projected prevalence as 55 percent.

The intervention site had the larger sample size (245) of the two areas. Thus, for the estimation of prevalence we needed at least 245 MWRA in each area. Among associated factors for intervention status, we determined the maximum sample size of 255 MWRA to detect an odds ratio of 2.5 at a significance level of.05 and study power of 0.8 when the team took proportion of females with antenatal care in non-intervention area as 25 percent [17].

Our calculations determined a larger sample size for associated factors, 255, as compared to the sample size for the estimation of prevalence, 245. Eventually, we took the larger sample size of at least 255 MWRA as required for estimation of prevalence as well as identification of associated factors in the two areas.

Finally, we inflated this sample size first for design effect, taking this as 1.68 (*roh*  =  Rate of homogeneity  =  0.02 and b  =  Average responses per cluster, 35 subjects per cluster) and for non- response, by 10 percent. The final sample size was taken as 472 for each of the two areas.

#### Sampling Strategy

To achieve the required sample size of 472 from each area, we selected 14 clusters (villages) from each area and from each cluster 35 MWRAs were interviewed. We selected these clusters by using the sampling technique of Probability Proportional to Size (PPS) [15]. In this way the research team selected all required clusters, with larger clusters having a higher probability of selection.

The field teams selected households in a cluster by systematic sampling with a random start, if there were more than one MWRA, one was selected through simple random sampling.

Selection of Clusters (Villages): Villages are informal geographic boundaries of varying sizes based on population and geographic spread. We took one village as a cluster.

Inclusion criteria: The research team included all villages, in intervention and control areas (the terms non- intervention and control areas are used interchangeably), in which project work started not later than October 1999 in the MDM Project and out of the Project area respectively.

Exclusion criteria:

- Villages with insufficient population to capture a cluster size. As our target in each cluster was 35 subjects (MWRA), for randomization purposes we excluded those villages which had less than 70 households. The study team assumed that each household would have at least one MWRA.
- We also excluded those villages in the intervention area which had some contamination with intervention components not being evaluated.

Indicators used: To measure the utilization of different components of MNH Care for the evaluation of maternal health programs, WHO, UNFPA, and UNICEF have recommended various indicators from which we chose the following [18].

1. Proportion of women receiving antenatal care;
2. Proportion of women with tetanus immunization;
3. Proportion of births conducted at health care facility; and
4. Percentage of women using contraceptive methods.

### Questionnaire

Firstly, we prepared our questionnaire in English, then translated into Urdu (the more common official language in local use), then back-retranslated into English for verification. Two independent professionals, not known to each other, performed this task. Following this we field-tested the questionnaire twice after three days of interviewer training. Then the field team under the supervision of the principal investigator (first author) finalized and administered the questionnaire in accordance with the sampling strategy. The research team obtained ethical approval from the Ethical Review Committee (ERC) of Aga Khan University (AKU) and field teams obtained written informed consent from study participants before the start of the interview. For minor participants in the study, written informed consent was taken from parents or guardians on the behalf of the minors/children participants.

At the study site, the most frequently spoken language is *Saraikie.* In principle, it would have been better to translate the final questionnaire to *Saraikie.* However, this was impractical because writing and reading of *Saraikie* language is very difficult even for native speakers. To minimize any linguistic gaps which may have remained, we trained interviewers to speak in *Saraikie* whenever they found a *Saraikie* speaking respondent.

The interview lasted for about 45 minutes. The questionnaire had different sections to obtain information on: socioeconomic and demographic characteristics, like: age of mother, parity, family size and education; antenatal care, such as: gestational age at first antenatal visit, type of health care provider visited, vaccination for tetanus, diet during pregnancy and taking any supplements; perinatal care, for example: place and mode of delivery, pregnancy outcome and cord care; postnatal care which included first feed to newborn, breastfeeding, any health related problems, visit to a health care provider. It was a structured questionnaire with some questions asked as open ended.

To monitor the quality of the information collected, the interviewers were accompanied by three field supervisors who selected the sampled households. Questionnaires were field edited for any missing and inconsistent information which was corrected after discussing with the interviewers. Final editing of the questionnaires was done after the completion of the interviews.

### Data Management

Field teams completed the data collection task during September through November 2001, under direct supervision of the first author. To maintain the quality of data collection, supervisors checked and edited the completed questionnaires in the field. The first author along with a senior field supervisor finally edited all the questionnaires before the data entry process. The data management team used EPI INFO version 6.04 for double entry of data from questionnaires and the first author performed analysis in SPSS software version 10 [19], [20].

#### Statistical Analysis

Descriptive analysis was done for the two groups separately. Means of the two groups were compared using independent t-tests for the continuous variables while proportions were compared for categorical variables using chi square tests or Fisher’s exact test. To look for the statistical difference between the two groups p-values have been provided.

We took intervention status as the dependent variable. Since multiple activities took place in the intervention area and we wanted to look for association of these factors with the intervention area, taking intervention status as the dependent variable and looking for the simultaneous effect of other variables with the intervention appeared more logical. For the purpose of analysis, we took non-intervention  =  0 and intervention  =  1.

Logistic regression was carried out to look for associated factors. As a first step, univariate analysis was done to look for the association of each independent variable individually. For each associated factor, an odds ratio and a 95% confidence interval was calculated by univariate analysis having that single independent variable. The likelihood ratio test was used for testing the significance of the coefficients. Independent variables with p-value of less than 0.25 were selected for the multivariate analysis. Moreover, the univariate analysis was divided in three groups i.e. antenatal, natal and postnatal period.

Multivariate analysis was performed to study the association of independent variables with the intervention while adjusting for the confounding effect of the other variables. In addition to the independent variables selected for univariate analysis, biologically plausible variables were also selected for multivariate analyses. Significance of each independent variable in the multivariate analysis was assessed by its confidence interval and Wald statistic. Variables, which were not significant at a p-value of 0.05 or not considered biologically plausible were removed from the model. The overall significance of the variables in the model was assessed by the G statistic.

Confounding was assessed by change in estimate of coefficient of 15%. At this stage multi- colinearity was checked and none of the variables was found showing high correlation with other variable. Thus the final model was completed. After the final model, plausible interactions were assessed. Adjusted odds ratios and 95% Confidence Intervals were used for interpretation of the model.

## Results

### Descriptive Analysis

We interviewed a total of 924 MWRA in the two areas, 432 in the intervention area and 492 in the non-intervention area.

#### Socio-demographic indicators

Mean age of women in both the areas (27.5 vs. 27.6; p =  0.995) as well as mean age of husbands (32.7 vs. 32.5; p =  0.982) were similar as shown in table 1. Education level was invariably higher in the intervention area for both women and husbands for most categories and it was significantly higher (p<.0004 and p<.0007 respectively). Similarly, a higher percentage of households in the intervention area with better household amenities indicated that families there were economically better off (p<.021). On the other hand, more households in the control area had electricity connections (84% vs. 79%) while *Saraikie was* the most frequently spoken language in the control area (97% vs. 74%) suggesting a culturally more diversified population in the latter.

**Table 1:** Socio-demographic characteristics of MWRA in Tehsil Khan Pur (September 2001).

Variables	Intervention (n = 432)	Non-intervention (n = 492)	p-value
	Frequency (%)	Frequency (%)	
**Age of woman (in years)**			.99
15–29	272 (63.0)	311 (63.21)	
30–39	136 (31.48)	154 (31.30)	
40 and above	24 (5.55)	27 (55)	
Mean	27.58*	27.51	
(SD)	6.01	6.07	
**Age of husband (in years)**			.07
15–29	124 (28.7)	136(27.64)	
30–39	233 (53.94)	270 (54.88)	
40 and above	76 (17.59)	124 (28.70)	
Mean	32.47**	32.69	
(SD)	7.05	7.27	
**Woman education**			.0004
Illiterate	344 (79.6)	437 (88.8)	
Primary	41 (9.5)	34 (6.9)	
Middle	18 (4.2)	10 (2.0)	
Secondary	29 (6.7)	11 (2.2)	
**Husband education**			.0056
Illiterate	207 (47.9)	292 (59.3)	
Primary	61 (14.1)	51 (10.4)	
Middle	57 (13.2)	47 (9.6)	
Secondary	107 (24.8)	102 (20.7)	
**Woman working for cash or kind**	207 (47.9)	278 (56.5)	0.011
**Husband profession**			.114
Farmer	84 19.4)	95 (19.3)	
Laborer	152 (35.2)	191 (38.8)	
Shopkeeper	48 (11.1)	53 (10.8)	
Private employee	36 (8.3)	19 (3.9)	
Government employee	26 (6.0)	29 (5.9)	
Businessman	21 (4.9)	20 (4.1)	
Landlord	57 (13.2)	67 (13.6)	
Retired/unemployed	8 (1.8)	18 (3.6)	
**Mother tongue**			
Saraikie	318 (73.6)	476 (96.7)	
Punjabi	110 (25.5)	11 (2.2)	
Others	4 (1.0)	5 (1.0)	
**Households with Electricity**	339 (78.5)	417 (84.8)	0.017
**Connection**			
**Ownership**			.021
Radio/cassette player	127 (29.4)	117 (23.8)	
T.V. Refrigerator	127 (29.4)	109 (22.2)	
Washing machine	67 (15.5)	50 (10.2)	
Motor bike/scooter	70 (16.2)	64 (13.0)	
Tractor	40 (9.3)	30 (6.1)	
Live stock	47 (10.9)	25 (5.1)	
House	290 (67.1)	310 (63.0)	
Land	417 (96.5)	480 (97.6)	
	218 (50.5)	230 (46.7)	
**Number of women**	432	492	

* p.995, **p.982.

#### Utilization of MNH services

Table 2 depicts the utilization of MNH services. Women in the intervention area utilized all components more frequently than those in the control area. Less than half of the women received antenatal care in the intervention area and about one-fourth of the women in the non- intervention area used these services (p<.000). More than two-fifths of the women received TT vaccination in the intervention area (42%), compared to less than one-third of the women in the non-intervention area depicting significant difference (p<.05). Similarly, a higher proportion of deliveries were conducted at health facilities in the intervention area (p<.0122), as well as use of modern contraceptive methods was also higher in the intervention than in the non-intervention area (p<.0202).

**Table 2:** MNH services utilization by MWRA in Tehsil Khan Pur (September 2001).

Variables	Intervention (n = 432)	Non-intervention (n = 492)	p-value
	Frequency (%)	Frequency (%)	
**Antenatal care received**	189 (43.8)	132 (26.8)	<.000
**TT Vaccination**	185 (42.8)	148 (30.0)	<.000
**Delivery at a health facility**	74 (17.1)	56 (11.4)	<.0122
**Contraceptive prevalence rate**	52 (12.0)	37 (7.5)	<.0202
**(Modern Methods)**			

### Univariate analysis

We carried out univariate analysis for three different periods: antenatal, perinatal and postnatal. We looked for association of the dependent variable with important and plausible independent variables among each of these periods. Variables from three phases of gestational life were finally selected for inclusion in the multivariate analysis, based on biological plausibility or p- value < 0.25.

As shown in table 3, the intervention area reflected healthy care seeking behaviors by respondents. Not only were women in the intervention area twice as likely to receive ANC (95% CI: 1.6–2.8) but they were even more likely to start ANC in the first trimester (OR = 2.5; 95% CI: 1.8–3.6). Respondents in the intervention area also exhibited positive practices regarding their nutrition and use of supplements; a significantly higher number of women increased their diet (OR = 1.8; 95% CI: 1.2–2.0) as well as consumed hemoglobin promoting supplements (OR = 1.8; 95% CI: 1.1–3.1).

**Table 3:** Univariate analysis of potential factors associated with intervention in rural areas of Tehsil Khan Pur with odds ratios and 95% confidence intervals (September 2001).

Variable	Intervention (n = 432)	Non-intervention (n = 492)	Unadjusted OR (95%CI)
ANTENATAL CARE			
**Antenatal care received**			
Yes	189	132	2.1 (1.6–2.8)
No	243	360	1.0
**_Start of antenatal care_**			
_1_^st^ trimester	104	61	2.5 (1.8–3.6)
_2_^nd^ trimester	38	28	2.0 (1.2–3.4)
3^rd^ trimester	47	43	1.6 (1.0–2.5)
No visit	243	360	1.0
**TT vaccination**			
Yes	239	170	2.3 (1.8–3.1)
No	193	322	1.0
**Perceived increase in diet**			
**in pregnancy**			
Yes	200	178	1.5 (1.2–2.0)
No	232	314	1.0
**Hematinics use during pregnancy**			
Yes	141	128	1.8 (1.1–3.1)
No	27	44	1.0
**Place of antenatal checkup**			
BHU	67	17	5.3 (2.9–9.8)
RHC	45	8	7.6 (3.4–17.1)
THQH	8	14	0.8 (0.3–1.9)
Private Clinic	69	93	1.0
**Antenatal care provided by**			
Dai	5	7	0.6 (0.2–1.8)
LHW	33	6	4.3 (1.7–10.6)
Skilled Birth Attendant	151	118	1.0
(Doctor/LHV)			
**Antenatal care motivator**			
LHWs	102	4	61.9 (16.4–234.5)
Doctor/LHV	14	20	1.7 (0.6–5.2)
Self-referral/family	64	85	1.8 (0.7–4.7)
Dai	7	17	1.0
**Knowledge of danger signs in**			0.9 (0.86–0.97)
**Pregnancy** *			
**DELIVERY CARE**			
**Place of delivery**			
Home	336	404	0.6 (0.4–0.9)
Hospital	74	56	1.0
**Attendant at delivery**			
LHW	9	2	5.5 (1.2–25.2)
Skilled Birth Attendant	75	57	1.6 (1.1–2.4)
Dai	326	401	1.0
**Material applied to umbilical**			
**cord stump**			
Nothing	46	36	1.2 (0.5–2.8)
Ghee/Oil	314	399	0.7 (0.3–1.6)
Animal dung	14	2	6.5 (1.2–34.2)
Antimicrobials	14	13	1.0
**Knowledge of danger signs in**			1.007 (1.004 –1.010)
**delivery** *			
**POSTNATAL CARE**			
**Breastfeeding**			
Yes	409	454	1.5 (0.9–2.5)
No	23	38	1.0
**Weighing of newborn**			
Yes	24	8	3.5 (1.5–7.8)
No	384	445	1.0
**Current use of contraceptive**			
**methods**			
Yes	52	32	1.7 (1.1–2.6)
No	380	455	1.0

* Taken as a continuous variable.

Delivery care also showed healthy practices; comparatively fewer women from the intervention area gave birth at home (OR = 0.6; 95% CI: 0.4–0.9). Moreover, a higher number of women were attended by a skilled birth attendant (SBA) at delivery (OR = 1.6; 95% CI: 1.1–2.4). However, in the intervention area, newborn care reflected dangerous practices as more newborn umbilical cord were covered with animal dung (OR = 6.5; 95% CI: 1.2–34.2).

Postnatal period practices were not much different between the two areas, except for current use of contraceptives which was higher in the intervention area (OR = 1.7; 95% CI: 1.1–2.6). The opinion of the respondents regarding the approval of husband for using contraceptive methods revealed mixed comments; relatively more women from the non-intervention area opined disapproval from their husbands. Knowledge of women regarding danger signs related to all three phases of gestational period was either poor or non-significant in the intervention areas.

### Multivariate analysis (Table 4)

**Table 4:** Multivariate analysis of factors associated with MNH interventions in rural areas of Tehsil Khan Pur in September 2001 with odds ratios and 95% confidence intervals (intervention  =  432, non-intervention  =  492).

Variables	*Adjusted OR (95%CI)
**ANTENATAL CARE**	
**Place of Antenatal check up**	
BHU	7.3 (3.6–15.1)
RHC	10.3 (4.2–25.2)
THQH	0.7 (0.2–1.8)
Private clinic	1.0
**TT Vaccination**	
Yes	1.9 (1.04–3.6)
No	1.0
**DELIVERY CARE**	
**Knowledge of danger signs in delivery**	1.01(0.99 – 1.01)

* These estimates were obtained after controlling for sociodemographic factors: education, profession, and economic status.

(household amenities) and distance of residence from the health facility.

Earlier studies have shown strong associations between MNH utilization and factors such as: education, occupation, economic status (measured through household amenities) [4], [21]–[27], and residential distance from health facility [5]. We therefore controlled for these variables to explain the utilization of reproductive health services.

While adjusting for other variables in the model, in the intervention area, BHU as the facility used for antenatal checkup was seven times more likely than in the non-intervention area (95% CI: 3.6, 15.1). Women in the intervention area were twice as likely to receive vaccination against tetanus (95% CI: 1.03, 3.5), while intervention associated with knowledge of danger signs in delivery was not statistically significant. We tested the final model for goodness of fit (Hosmer and Lemeshow’s test) and found fit (p = 0.721).

## Discussion

Improved MNH service utilization was demonstrated in the intervention area: a significantly higher proportion of women received antenatal care, received TT vaccination, delivered at a health facility and used modern contraceptive methods.

The project mostly used face-to-face awareness creation sessions by LHW and community mobilization by VHCs. The research evidence from Pakistan [10], [11], [28], [29] and other developing countries [3], [30] shows that increasing awareness at the family or community level through individual sessions of community worker with the community member or through group activities results in change in health seeking behavior. A community-based newborn care project reported increased institutionalized deliveries; their intervention package contained training of LHWs and *dais* as well as arranging for group sessions at community level [29].

Similarly, Balochistan Safe Motherhood Initiative (BSMI) and Safe Motherhood Applied Research and Training (SMART) project also used both the strategies for increasing awareness and mobilization in the community [10], [11]. Findings from BSMI suggested a significant increase in the proportion of women seeking antenatal care (ANC), TT vaccination and delivering at a health facility [10]. Population Council, Pakistan, introduced a client centered approach in its SMART project in addition to arranging LHWs training and support group sessions; at the endpoint, the project findings suggest an increased proportion of women going for ANC and receiving TT vaccination [11]. Findings from Tanzania and Burkina Faso also show that community workers play a significant role in improving the utilization of health services [3], [30]. These projects had also utilized face-to-face meetings and group sessions for awareness creation.

Similar to our study, but with more programmatic focus on training of LHWs for individual sessions, an overall evaluation of the national LHWP by Oxford Policy Management (OPM) reported a comparatively higher proportion of women contacted by LHWs sought ANC as well as received TT vaccination (28). Both strategies have their advantages and disadvantages. Face-to-face sessions may be time consuming but ensure privacy and provide an opportunity for clients to discuss personal issues which they may not wish to discuss in group sessions. On the other hand, group sessions save time and more community members can obtain knowledge; at the same time peer pressure may also motivate them towards healthy behaviors. However, arranging group sessions and bringing people to one place during specific intervals, sometimes becomes a significant logistical challenge.

In the final logistic regression model, all the variables which were found significantly associated with the intervention area at univariate level lost association except place of ANC and TT vaccination. The location of the ANC was found to be statistically significant. The RHC and BHU were used more frequently by women for their antenatal check up in the intervention area.

It has been suggested that increased utilization of health services may reflect good quality of care, being an outcome of the performance of a system to provide care [31]. Level of necessary knowledge, required skills of care providers and adequate supplies together with Interpersonal Communication (IPC) also affect care processes. This has been explored in rural areas of Burkina Faso where a study revealed underutilization of services related to pregnancy and delivery care because of lack of supplies and equipment, inadequate knowledge and skills of care providers [32].

In the intervention area, at a rural health center, a skilled birth attendant (SBA), midwife/LHV provides services for 24 hours backed up by an on-duty medical doctor who is also on call between working hours. This team is well equipped with ultrasonographic facilities along with a small laboratory for basic blood and urine tests and a well-established labor room. The team is well trained in IPC skills. The literature from other south Asian settings also supports this approach: in Sri Lanka, the presence of increased number of well-trained staff with supplies had a high probability of utilization [33]. Results of a study in rural Nepal revealed four to six times greater use of facilities with high quality antenatal services as compared to institutions offering low quality care [6].

Our data suggest very low utilization of the THQH for antenatal services in both areas. Large hospitals outside the immediate locality of a client are less likely to be utilized by rural residents as the environment is unfamiliar and access is not easy. This is consistent with the model, “the need for the familiar” [34], and could explain why clients prefer the BHU and avoid THQH as the former is in their locality while the latter requires orienting to a more urban environment.

Being a referral hospital, the turnover of patients at the THQH is high. Providers often give very limited attention to their clients which may greatly affect the provider-client interaction. At most secondary care hospitals (hospitals with specialists as well as inpatient facilities like THQH and District Headquarters Hospitals) due to high turnover of clients, the provider-client interaction may not be favorable. Evidence has shown that poor provider-client interaction is a strong determinant of underutilization of health services [33]. The behavior of clients is very complex when it comes to the quality of services and utilization: people, irrespective of economic status, may choose services with high quality and by-pass nearby poor quality health facilities for distant facilities with relatively better services ignoring the cost of time and money [33], [35].

To improve the MNH indicators in rural Pakistan, availability of appropriate services and their timely utilization play a pivotal role. Community health workers, by providing essential knowledge to the community and referring them to health facilities equ ipped with essential staffing and supplies, can have beneficial effects.

At univariate level, the findings do not show any difference in breastfeeding patterns between the two areas, although a higher proportion of the respondents in the intervention area breastfed their newborns. In these rural areas, usually most families breastfeed as a cultural practice consistent with religious belief. Moreover, breastfeeding helps reducing the cost of bottle feeding as well as the additional task of preparing feed at regular intervals, which are good incentives for rural families living with limited resources.

These estimates regarding knowledge about danger signs of pregnancy, delivery and postpartum period lost association at multivariate level except for danger signs of delivery which was marginally significant. Similar findings were found at the endpoint survey of a community based project implemented in a similar setting [36]; it did not show much difference between intervention and control arms in the knowledge of dangers signs of delivery and postpartum period but was significantly higher for danger signs in pregnancy. One reason could be that this project had hired and trained community mobilization staff solely for this purpose and they had utilized the support group strategy in a majority of areas. While in our study LHWs were the health educators, they were performing this task in addition to other responsibilities and this increased work burden may have compromised their health education task.

The univariate analysis also reflected husbands’ disapproval for the contraceptive use. However, this opinion question was asked from participants regarding the husbands’ decision; this factor lost its association at multivariate analysis. Nonetheless, the contraceptive prevalence rate (CPR) which is the output indicator for contraceptive education was significantly higher in the intervention area, which is consistent with an LHW educational effect.

This midterm evaluation of the Women’s Health Project implemented by MDM suggests favorable changes in the reproductive health behavior of the women (which may also reflect behavior of families and communities at large) in core areas of maternal health such as, antenatal care practices including antenatal care visits; better diet and nutrition; TT vaccination, institutional deliveries or deliveries attended by SBAs and higher use of contraceptive methods. Some areas still need more emphasis, especially knowledge about danger signs of pregnancy, delivery and postnatal period as well as newborn care, especially cord care.

The recommendations of the Mid-term Evaluation (MTE) were implemented in the project site. To address the sustainability issue, MDM gradually transferred the responsibility for the project to a local NGO: the Maternity and Child Welfare Association Khan Pur (MCWAK). After two years of joint management, MDM withdrew from the site in 2004. Since then, MCWAK has expanded the intervention package, as well as the catchment population. In 2006, MCWAK set up a community midwifery school. In 2013, MCWAK is running three mother and child health clinics.

## Limitations

Cross sectional study design does not necessarily support any causal linkage between intervention and the outcome. No formal baseline survey was conducted before starting this project, which would have provided opportunities to make comparisons between the findings of the baseline and the endpoint surveys. Such comparisons would also have supported the causal link between intervention and outcome. Besides, at the time of this mid-term review various types of intervention activities were passing through different phases of implementation and may have resulted in different levels of their effect on the outcome; however, every effort was made to include those intervention activities which were in place for a sufficient period of time.

## Conclusions

Despite the overall success achieved so far in response to most of the Millennium Development Goals, over the past two decades decreases in maternal mortality are far from the 2015 target for MDG-5, globally in general and in Pakistan in particular [1]. The evaluation reported here sheds light on the barriers to improving reproductive health, while illuminating key success factors, especially the role of LHWs in encouraging use of modern contraception methods, improving antenatal care, achieving safer deliveries and promoting safer practices during the postnatal period. This evaluation serves as an interim measure of a national and global challenge that remains a work in progress.
